# Trends in Non-Tuberculous Mycobacterial Lung Disease and Treatment Outcomes in a Low-Tuberculosis Prevalence Setting: A Retrospective Analysis

**DOI:** 10.3390/pathogens13040344

**Published:** 2024-04-22

**Authors:** Biplob Kumar Mohanty, Tomas Mikal Lind Eagan, Bernt Bøgvald Aarli, Dag Harald Skutlaberg, Tehmina Mustafa

**Affiliations:** 1 Centre for International Health, Department of Global Public Health and Primary Care, University of Bergen, 5020 Bergen, Norway; biplobmohanty89@gmail.com; 2 Department of Thoracic Medicine, Haukeland University Hospital, 5021 Bergen, Norway; tomas.eagan@uib.no (T.M.L.E.); bernt.aarli@uib.no (B.B.A.); 3 Department of Clinical Science, University of Bergen, 5020 Bergen, Norway; 4 Department of Microbiology, Haukeland University Hospital, 5021 Bergen, Norway

**Keywords:** mycobacterium avium, non-tuberculous mycobacteria, treatment response, relapse

## Abstract

Background: Information on the management of non-tuberculous mycobacterial (NTM) lung infection and disease is scarce. The aim of this study was to investigate the trends in NTM lung infections, and the factors associated with the initiation of treatment and treatment outcomes. Methods: A retrospective analysis was carried out on patient medical records from Haukeland University Hospital, Bergen, Norway, from 2000 to 2021. Results: Among 154 patients with NTM lung infection, the majority (70%) were older than 65 years, and 49% had an underlying pulmonary comorbidity. The most frequently observed mycobacterial species was *M. avium* complex (MAC), followed by *M. malmoense* and *M. abscessus*. In total, 72 (47%) patients received antibiotic treatment. Patients with high symptom scores, aged below 65, and with MAC infection had more than three times the odds of receiving antibiotic treatment. A favourable response and culture conversion was observed in 53 of 72 (74%) patients. However, 17 (32%) of them had a relapse. Out of 82 patients who did not receive treatment, 45 (55%) had spontaneous culture conversion, and 8 (18%) of them had a relapse. No factor was identified to be significantly associated with a favourable treatment response. Conclusion: A favourable response to treatment was seen in 74% of patients with a high relapse rate.

## 1. Introduction

Non-tuberculous mycobacterial (NTM) infections are a public health problem worldwide. Despite being overshadowed by infections from other members of the *Mycobacteriaceae* family, such as *Mycobacterium tuberculosis* and *Mycobacterium leprae*, NTM infections are associated with considerable mortality and morbidity [1,2]. The incidence of NTM infections is particularly high in the elderly population, and it is projected to continue to rise in the coming years due to the increasing number of elderly individuals [3].

NTM infections can cause a range of diseases, including NTM lung disease (NTM-LD). Some of the more common NTM species known to cause NTM-LD are *Mycobacterium avium complex* (MAC), *Mycobacterium kansasii*, *Mycobacterium xenopi*, *Mycobacterium abscessus,* and *Mycobacterium malmoense* [4]. These infections typically occur as comorbidities in patients with underlying respiratory diseases such as chronic obstructive pulmonary disease (COPD), bronchiectasis, and cystic fibrosis (CF) [5]. However, the diagnosis of NTM infections can be challenging, and patients often face a lengthy time to diagnosis or misdiagnosis, leading to a poor long-term outcome. Information on the prevalence of NTM infections is usually lacking, as NTM infections do not require official notification. However, there is a need to investigate the trends and characteristics of NTM infections in specific geographic areas to provide information for timely diagnosis and treatment.

This study investigated the trends and characteristics of NTM lung infections and NTM-LD over the past two decades at a tertiary care hospital in western Norway. We also aimed to identify factors associated with the initiation of treatment for NTM-LD and treatment outcomes.

## 2. Materials and Methods

A retrospective analysis of the patients’ medical records was carried out at Haukeland University Hospital, Bergen, Norway. All inpatient and outpatient records between 2000 and 2021 were searched for the International Classification of Diseases, 10th Revision (ICD-10) code A31, including A31.0, A31.1, A31.8, and A31.9, to identify eligible cases. Patients diagnosed with NTM lung infection and NTM-LD who met the current American Thoracic Society (ATS) criteria were included in the study [4].

**Figure 1:**
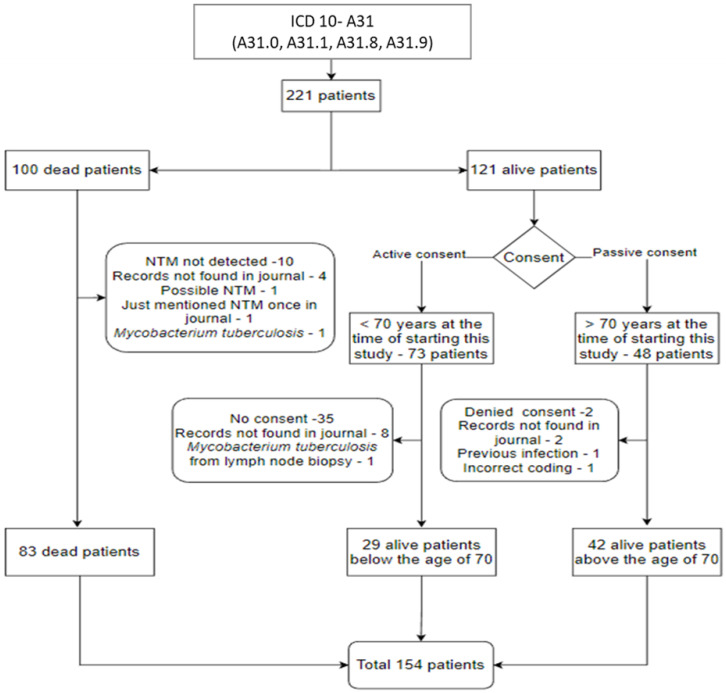
Flow chart showing the inclusion and exclusion of patients in the study.

Figure 1 presents the selection process for the study sample. Two hundred and twenty-one potential patients with NTM lung infection were identified by having at least one record coded with A31. Among the 100 deceased patients, records with confirmed NTM lung infection were available for 83 patients. Thirty-five patients who were alive and under 70 years old did not provide consent and were excluded. For alive patients above the age of 70, 42 records were available. The final study sample included 154 patients.

### 2.1. Data Collection

Relevant data were collected from the electronic medical records of the Department of Thoracic Medicine, Haukeland University Hospital. The variables collected included sex, age, presence of pulmonary comorbidities, symptoms, microbiological findings, radiological findings (both chest X-ray and chest CT scan), treatment history, and treatment outcome. Six symptoms of particular interest were recorded: fever, cough, haemoptysis, night sweats, dyspnoea, and weight loss.

### 2.2. Definitions

A symptom score was calculated, which was defined as a sum of the total number of symptoms where a score of 1 was given for each symptom. A score of 3 or more was considered a high symptom score. A favourable response to treatment or culture conversion was defined as at least two consecutive negative mycobacterial cultures from respiratory samples.

NTM lung infection was defined as a positive culture for NTM from two separate expectorate sputum samples or positive culture from one bronchoalveolar lavage but with the clinical or the radiological criteria for the NTM-LD unfulfilled. NTM-LD was defined as NTM lung infection and the presence of symptoms and nodular or cavitary opacities on chest X-ray or chest computer tomography (CT) scan.

### 2.3. Data Management and Analysis

The collected data were analysed using IBM SPSS statistics software version 27. Binary logistic regression was used for univariate and multivariate analyses to identify factors associated with the initiation of antibiotic treatment and factors associated with treatment outcome. The unadjusted odds ratio (OR) and 95% confidence interval (CI) for each variable were first calculated using univariate regression. Multivariate regression was then performed. All variables were included in the final model. The adjusted OR (aOR) and 95% CI were calculated for each included variable. A *p*-value ≤ 0.05 was considered statistically significant.

### 2.4. Ethical Clearance

Ethical clearance was obtained from the Regional Committee for Medical Ethics Western Norway, reference number 282165. Active consent was obtained from patients below the age of 70, while passive consent was taken from patients above the age of 70. In the case of deceased patients, consent was not required. All data were de-identified to protect patients’ privacy (i.e., social security numbers, names, or other directly identifiable characteristics were removed), in accordance with local regulations.

## 3. Results

Of the 154 patients, 75 and 79 fulfilled the criteria for NTM lung infection and NTM-LD, respectively. The demographic and clinical characteristics of patients in the two groups did not vary significantly and are presented together in Table 1. Seventy percent of the study sample were patients older than 65 years. The most reported symptoms were cough (82%), dyspnoea (43%), and weight loss (33%). All the patients had pathological radiological features that varied depending on the underlying pulmonary comorbidity, including nodular opacities, opacities with tree-in-bud appearance, atelectasis, and emphysema. Pulmonary cavities were observed in 25% of the patients. The majority (62%) had pulmonary comorbidities, 30% had COPD, 19% bronchiectasis, and 13% had both COPD and bronchiectasis. A total of six main groups/species causing NTM infections were identified, including *M. avium complex* (MAC), *M. malmoense*, *M. abscessus*, *M. gordonae*, *M. fortuitum*, and *M. xenopi*.

**Figure 2:**
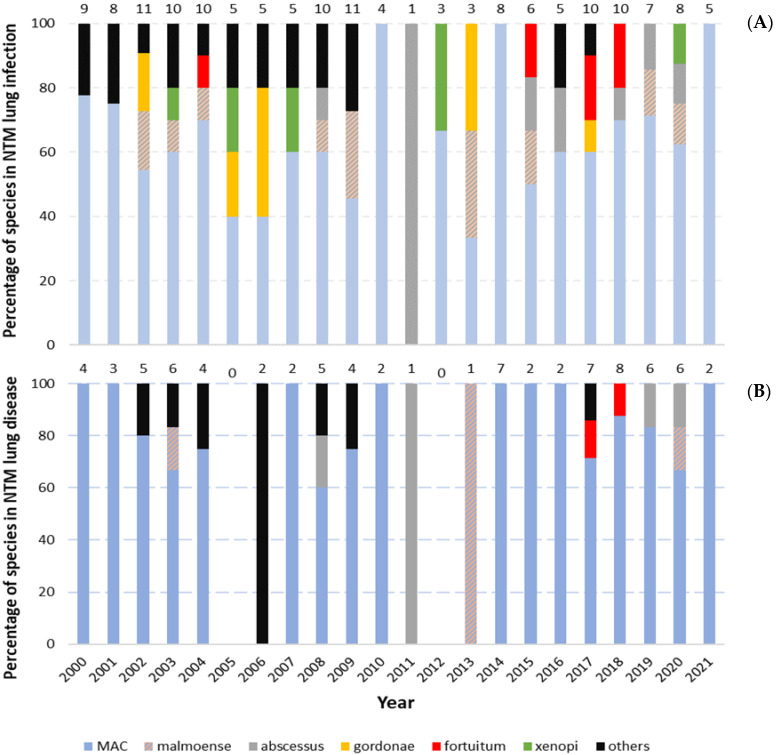
(**A**,**B**) Proportion of different species in NTM lung infection and NTM lung disease from the year 2000 to 2021. Total amount of NTM lung infections and NTM lung disease for each year shown at the top of the bars.

The proportion of different NTM species in NTM lung infections and NTM-LD over the past 22 years is presented in Figure 2A and Figure 2B, respectively.

**Figure 3:**
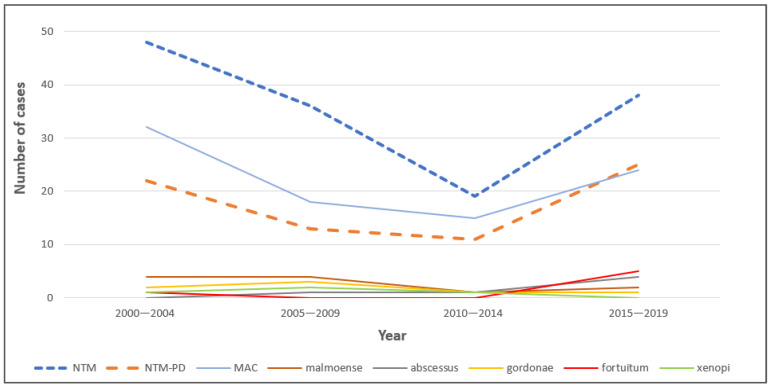
Trends in different species in NTM lung infection in 5 year periods from the year 2000 to 2019.

Figure 3 shows that the number of cases with NTM lung infection declined every five years, with 48 (31%), 36 (23%), and 19 (12%) cases for the time periods 2000–2004, 2005–2009, and 2010–2014, respectively. The number of cases with NTM infection increased to 38 (25%) in 2015–2019. The trend for NTM-LD followed a similar pattern. All species followed a similar trend across the years.

Table 2 shows the clinical characteristics of patients with NTM lung infection and NTM-LD caused by different species. All species were associated with low symptom scores and predominantly non-cavitary disease. Apart from *M. gordonae*, infections with all species received treatment. All species showed a positive response to treatment in more than 60% of the cases, except for *M. malmoense*, where a positive response to treatment was shown in only 25% of cases. In *M. malmoense* infections, relapse was seen in 100% of cases. A total of 71% of patients with *M. abscessus* infection, 57% of patients with MAC infection, 40% of patients with *M. xenopi* infection, and 33% of patients with *M. malmoense* and *M. fortuitum* received treatment.

Table 3 shows the factors associated with the initiation of antibiotic treatment. Of the 154 patients with NTM lung infection, 79 patients fulfilled criteria for NTM-LD. Treatment was started for patients with infection and with disease. In all, 72 (47%) patients received antibiotic treatment. Of these, 49 patients had NTM-LD and 23 patients had NTM lung infection. Patients with high symptom scores, those below the age of 65, and those with MAC infection had more than three times the odds of receiving treatment (*p* = 0.006, *p* = 0.006, and *p* = 0.005, respectively). Thirty-one (56%) patients were started on treatment more than 6 months after diagnosis. The first guidelines on the treatment of NTM lung disease in 2007 did not impact the decision to start treatment.

### Data Reported as n (%)

Table 4 shows the combination of antibiotics used for the treatment of different species. The most common antibiotic regimen was macrolide (clarithromycin or azithromycin), rifampicin, and ethambutol. The duration of treatment with this regimen was 1 year after sputum conversion. The duration of treatment with linezolid, tigecycline, imipenem, and amikacin was shorter and varied among patients depending on the tolerance. A bronchial wash with hypertonic saline was used only by 27% (42/154) of patients. There was no significant difference in bronchial wash between patients who received antibiotics as compared to those who did not receive antibiotics.

Data reported as absolute numbers n of patients treated. Information was not available for 10 patients. Macrolide = Azitromycin or Clarithromycin.

Table 5 shows the factors associated with response to treatment. No factor was identified to be significantly associated with a favourable treatment response, including the time taken to start treatment or the presence of pulmonary cavities. There was no significant difference in response to treatment or relapse between NTM lung infection and NTM-LD.

**Figure 4:**
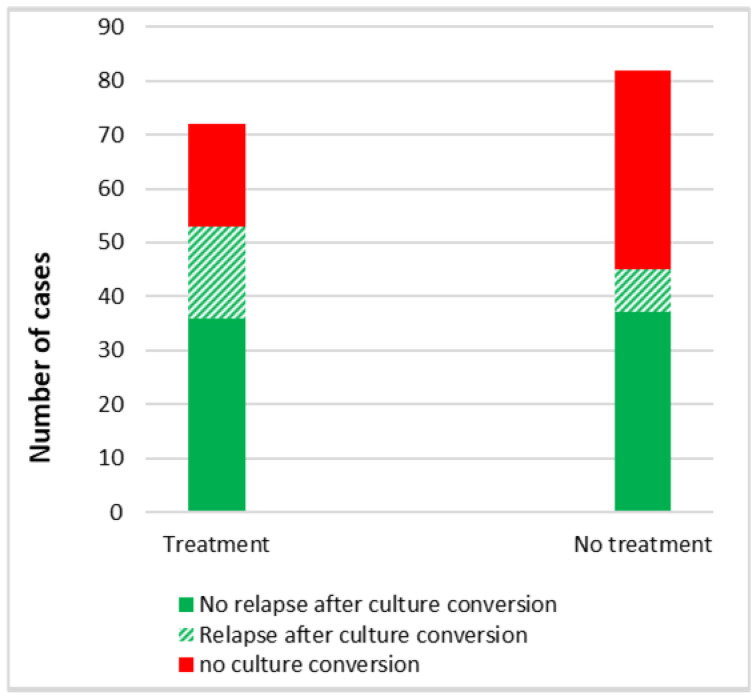
Comparison of culture conversion between patients with NTM lung infection according to their treatment status.

Figure 4 shows the comparison of culture conversion between treatment and non-treatment groups. Out of 72 patients who received treatment, 53 (74%) had a favourable response and had culture conversion; 17 (32%) of them had a relapse. Out of 82 patients who did not receive treatment, 45 (55%) had spontaneous culture conversion; 8 (18%) of them had a relapse. No factor was identified to be significantly associated with relapse.

## 4. Discussion

To our knowledge, this is the first study to investigate the trends in NTM lung infections and factors associated with the initiation of treatment and outcomes from a high-income and low TB-endemic setting of Western Norway over a period of 20 years. The major species observed were MAC, *M. malmoense*, and *M. abscessus*. The NTM infections showed a declining trend until 2014, followed by an increase in later years. This increasing trend could be attributed to an increased focus on the detection of mycobacteria from the respiratory samples. Furthermore, an increase in life expectancy with pulmonary comorbidities could also be a contributing factor, as indicated by an increased risk of contracting NTM infection with increased age and the presence of pulmonary comorbidity in the current study. Several studies have shown that age is a significant factor associated with the rise in incidence rates of NTM-LD among older populations, with a higher increase in the annual prevalence in individuals aged ≥60 years than in those aged <60 years [6,7,8]. Al-Houqani et al. [9] found that age is a significant contributor to the increase in incidence rates of NTM-LD, although it accounted for less than a quarter of the total increase. This suggests that other factors may also play a role in the rise of NTM-LD incidence rates. One such factor is chronic lung disease, including COPD, bronchiectasis, and cystic fibrosis, which are known predisposing factors for NTM-LD development [10]. In the present study, COPD was the most associated chronic lung disease (30%), followed by bronchiectasis (19%). Both diseases have increased in prevalence over the last 30 years and are themselves associated with higher age. COPD and bronchiectasis both disrupt the normal architecture of the airways, resulting in chronic bronchitis, with a disruption of the epithelial membranes and increases in mucus production [11]. This, unfortunately, is an ideal environment for bacterial colonization and overgrowth. In addition, several patients with COPD may have reduced general immunity due to age or poor nutrition. Patients with COPD are prone to develop cachexia [12]. Finally, many COPD patients use inhaled corticosteroids (ICS) to prevent COPD exacerbations. In some COPD patients, ICS treatment may also increase their risk of bacterial infections, including NTM-LD [13]. In cystic fibrosis, due to impaired mucociliary clearance, there are viscous airway secretions which predispose the patient to bacterial colonization and infection [14]. A systematic review and meta-analysis review of 95 studies have reported the prevalence of NTM infection in cystic fibrosis to be 7.9% and increasing over time [15].

In our data, 47% of patients received antibiotic treatment for the eradication of NTM. Patients with high symptom scores had greater odds of receiving treatment, and the odds of receiving treatment were much higher when the patient was below the age of 65. Interestingly, the presence of a cavity did not have a major impact on the initiation of treatment. However, a previous study has shown that cavitation on CT imaging, the presence of night sweats, and weight loss were associated with treatment initiation [16]. One study from South Korea demonstrated that the long-term treatment success rate decreased with age, and the rate of adverse drug reactions requiring a discontinuation of treatment increased with age, particularly in patients aged ≥80 years [17]. These findings support the decision to treat relatively younger patients, where better treatment outcomes are expected with fewer side effects. However, our study did not find that younger age is associated with a favourable treatment response.

In the present study, the odds of receiving treatment increased if the patient had MAC infection. As per the study, the principal causative species for NTM-LD were members of MAC. Several groups in multiple countries have documented the increasing incidence of MAC-related pulmonary disease [18]. Not all patients will require treatment initially, but most will during the course of the disease. Interestingly, the first guidelines on the treatment of NTM lung disease in 2007 did not impact the decision to start treatment.

In the present study, the patients who were initiated with treatment within 6 months or later did not differ in their time to culture conversion. One previous study [19] has noted that the waiting period between the diagnosis and treatment of NTM-LD patients did not impact culture conversion in patients. There are reports of patients with infection by *M. abscessus* or MAC and of elderly patients having associations with treatment failure [20]. However, in the current study, patients with MAC infection had higher odds of culture conversion. This could be due to the predominance of MAC species.

An interesting finding in this study was the spontaneous culture conversion in 55% of patients who did not receive antibiotics with a relative lower relapse rate, as compared to the higher proportion of culture conversion among 74% patients who received antibiotics but also with a relatively higher relapse rate. An earlier report has shown the elimination of MAC from the sputum after bronchial hygiene [21]. However, in our study, there was no significant difference in bronchial hygiene measures between patients who received antibiotics as compared to those who did not receive antibiotics. We could not identify any factors significantly associated with relapse.

The current study has some limitations. This is a retrospective study from a single hospital and some clinical data are missing. The study has a limited sample size, which may skew the results and factors toward MAC, as it constitutes the majority of NTM infections. Furthermore, 47% of the patients under 70 years did not give their consent and this could have skewed the results. Additionally, a small sample size may not provide an accurate representation of the trends. Another limitation is the accuracy of data input, as we obtained our data by reviewing patients’ journals. However, since a digital patient file system was first used in the early 2000s, data entry during the initial years may not have been complete, which could impact our findings. Therefore, we suggest that the results of this study need verification by large, multi-centre, prospective cohort studies.

## 5. Conclusions

Our study found that the major species causing NTM-LD in Western Norway from 2000 to 2021 were MAC, *M. malmoense*, and *M. abscessus*. Increased age and presence of pulmonary comorbidity increased the risk of contracting NTM lung infection. Patients with high symptom scores, ages below 65 years, and MAC infection had higher odds of receiving treatment. A favourable treatment response was seen in 74% of patients who received antibiotic treatment. Spontaneous remission was found in 55% of cases who did not receive antibiotics. Factors associated with favourable treatment response were not found. The results of this study need verification by large, multi-centre, prospective cohort studies.

## Figures and Tables

**Table 1:** Demographic and clinical characteristics of patients with NTM lung infection.

Characteristics	N = 154
**Sex**	
Male	71 (46%)
	
**The age range at the time of diagnosis in years**	
17–49	10 (6%)
50–65	38 (25%)
66–80	89 (58%)
81–94	17 (11%)
	
**Symptoms**	
Cough	126 (82%)
Dyspnoea	66 (43%)
Weight loss	51 (33%)
Haemoptysis	21 (14%)
Fever	16 (10%)
Night sweats	15 (10%)
	
**Radiological feature**	
Cavitary	39 (25%)
Pulmonary comorbidities	
COPD	46 (30%)
Bronchiectasis	30 (19%)
COPD + Bronchiectasis	20 (13%)
	
**Mycobacterial species**	
*M. avium complex (MAC)*	99 (64%)
*M. malmoense*	12 (8%)
*M. abscessus*	7 (5%)
*M. gordonae*	7 (5%)
*M. fortuitum*	6 (4%)
*M. xenopi*	5 (3%)
*Mixed infection*	4 (2%)
Others *	10 (6%)
Unknown **	4 (2%)
	
**Isolation of other respiratory pathogenic bacteria**	
*Haemophilus influenzae*	14 (9%)
*Staphylococcus*	13 (8%)
*Streptococcus*	10 (6%)
*Pseudomonas*	7 (5%)
*Mycobacterium tuberculosis*	5 (3%)
*Haemophilus parainfluenzae*	3 (2%)

* Two cases each of *M. chelonae* and *M. simiae*. One case each of *M. celatum*, *M. gadium*, *M. neoaurum*, *M. nonchromogenicum*, *M. peregrinum,* and *M. triplex*. ** NTM species not mentioned in the patient’s journal. Data reported as absolute numbers (%).

**Table 2:** Clinical characteristics of patients with NTM lung infection caused by different species.

Category		MAC	*M. malmoense*	*M. abscessus*	*M. gordonae*	*M. fortuitum*	*M. xenopi*	Mixed Infection	Others	Unknown
		(N = 99)	(N = 12)	(N = 7)	(N = 7)	(N = 6)	(N = 5)	(N = 4)	(N = 10)	(N = 4)
Age below 65		31 (31)	5 (42)	4 (57)	1 (14)	2 (33)	2 (40)	0 (0)	1 (10)	1 (25)
Male		43 (43)	7 (58)	1 (14)	4 (57)	3 (50)	2 (40)	1 (25)	6 (60)	3 (75)
Cavities		24 (24)	5 (42)	1 (14)	1 (14)	2 (33)	2 (40)	1 (25)	1 (10)	2 (50)
High symptom score		37 (37)	4 (33)	2 (29)	0 (0)	1 (17)	2 (40)	2 (50)	1 (10)	3 (75)
Treatment given		56 (57)	4 (33)	5 (71)	0 (0)	2 (33)	2 (40)	0 (0)	2 (20)	1 (25)
Positive response to treatment *		42 (75)	1 (25)	3 (60)	NA	2 (100)	2 (100)	NA	2 (100)	1 (100)
Relapse after treatment **		15 (36)	1 (100)	0 (0)	NA	0 (0)	1 (50)	NA	0 (0)	0 (0)
Spontaneous sputum conversion ***		23 (53)	6 (75)	1 (50)	3 (43)	0 (0)	2 (67)	3 (75)	6 (75)	1 (33)
Relapse after spontaneous sputum conversion ****		4 (17)	1 (16)	1(100)	0 (0)	NA	0 (0)	1 (33)	1 (17)	0 (0)

Data reported as n (%) percentage calculated by the formula n × 100/N. * N is equal to amount of antibiotic treatment given; ** N is equal to amount of positive response to treatment; *** N is equal to amount of antibiotic treatment not given; **** N is equal to spontaneous sputum conversion without antibiotic treatment.

**Table 3:** Factors associated with the initiation of antibiotic treatment for the eradication of non-tuberculous mycobacteria (NTM) among patients with NTM lung infection.

		Treatment n (%)	No treatment n (%)	Unadjusted Odds Ratio (95% CI)	*p*-Value	Adjusted Odds Ratio (95% CI)	*p*-Value
Total Patients (N = 154)		72	82				
Sex	Male	30 (42)	41 (50)	0.71	0.3	0.82	0.6
				(0.38–1.35)		(0.39–1.72)	
	Female	42 (58)	41 (50)	1		1	
Age	<65	30 (42)	15 (18)	3.19	0.002	3.18	0.006
				(1.54–6.62)		(1.4–7.21)	
	≥65	42 (58)	67 (82)	1		1	
Symptom score	SS 3–6	33 (46)	20 (24)	2.62	0.006	3.08	0.006
				(1.32–5.2)		(1.37–6.89)	
	SS 0–2	39 (54)	62 (76)	1		1	
Radiological feature	Cavitary	15 (21)	24 (29)	0.64	0.23	0.58	0.22
				(0.30–1.34)		(0.24–1.39)	
	Non–cavitary	57 (79)	58 (71)	1		1	
Pulmonary comorbidity	Yes	42 (58)	54 (66)	0.72	0.17	0.87	0.72
				(0.38–1.4)		(0.4–1.9)	
	No	30 (42)	28 (34)	1		1	
Microbiological species *	MAC	56 (79)	43 (54)	3.13	0.002	3.04	0.005
				(1.52–6.4)		(1.39–6.63)	
	Others	15/71	36/79	1		1	
		(21)	(46)				
Isolation of respiratory bacteria	Yes	24 (33)	20 (24)	1.55	0.11	1.51	0.32
				(0.77–2.13)		(0.67–3.39)	
	No	48 (67)	62 (76)	1		1	
Year of treatment	Before 2007	27 (38)	31 (38)	0.99	0.97	1.63	0.23
				(0.51–1.9)		(0.73–3.64)	
	In/after 2007	45 (62)	51 (62)	1		1	

* NTM species not known in 1 case where treatment had been given and in 3 cases where treatment had not been provided. Therefore, N = 71 and N = 79, respectively, for treatment and no treatment.

**Table 4:** Antibiotic combinations for the treatment of NTM lung infection according to the species.

Antibiotic Combinations	MAC (n)	*M. abscessus* (n)	*M. malmoense* (n)	*M. xenopi* (n)	*M. fortuitum* (n)
Macrolide + Ethambutol + Rifampicin	33		3		
Macrolide + Ethambutol + Isoniazid	3				
Macrolide + Ethambutol + Rifampicin + Isoniazid	3				
Macrolide + Ethambutol	4				
Ciprofloxacin + Ethambutol	1				
Rifampicin + Ethambutol	4				
Ciprofloxacin + Ethambutol + Rifampicin	2		1		
Ethambutol + Moxifloxacin	1				
Amikacin + Clofazimine + Moxifloxacin		1			
Macrolide + Amikacin + Cefoxitin + Bactrim		1			
Amikacin + Tigecyclin + Macrolide		1			
Amikacin + Tigecykline + Macrolide + imipenem + Linezolid		1			
Macrolide + Rifampicin + Ethambutol + Moxifloxacin				1	
Ciprofloxacin + Trimethoprim-Sulpha					1
Amikacin + Imipenem + Linezolid					1

**Table 5:** Factors associated with a favourable response to treatment among patients with NTM lung infection.

		Favourable Response to Treatment n (%)	No Response to Treatment n (%)	Unadjusted Odds Ratio (95% CI)	*p*-Value	Adjusted Odds Ratio (95% CI)	*p*-Value
Total patients (N)		53 (74)	19 (26)				
Sex	Male	21 (40)	9 (47)	0.73	0.56	0.85	0.78
				(0.25–2.1)		(0.27–2.64)	
	Female	32 (60)	10 (53)	1		1	
Age	<65	20 (38)	10 (53)	0.55	0.26	0.55	0.3
				(0.19–1.57)		(0.17–1.72)	
	≥65	33 (62)	9 (47)	1		1	
Symptom score	SS 3–6	25 (47)	08 (42)	1.22	0.7	1.16	0.8
				(0.43–3.54)		(0.36–3.72)	
	SS 0–2	28 (53)	11 (58)	1		1	
Radiological feature	Cavitary	10 (19)	5 (26)	0.65	0.5	0.63	0.49
				(0.19–2.23)		(0.16–2.39)	
	Non-cavitary	43 (81)	14 (74)	1		1	
Time taken for initiation of treatment *	≤6 months	23 (55)	8 (62)	0.69	0.31	0.73	0.42
				(0.34–1.41)		(0.34–1.56)	
	>6 months	19 (45)	5 (38)	1		1	
Pulmonary comorbidity	Yes	28 (60)	8 (53)	2.08	0.18	1.95	0.28
				(0.71–6.1)		(0.58–6.50)	
	No	25 (40)	10 (47)	1		1	
Hypertonic saline inhalation	Yes	19 (36)	7 (37)	0.96	0.94	0.77	0.67
				(0.32–2.84)		(0.23–2.54)	
	No	34 (64)	12 (63)	1		1	
Microbiological species **	MAC	42 (81)	14 (74)	1.63	0.42	1.69	0.43
				(0.5–5.29)		(0.46–6.13)	
	Others	10 (19)	5 (26)	1		1	

* Since information was not available for time to initiation of treatment in 17 patients, the value of N changed to N = 42 for favourable response to treatment and N = 13 for response to treatment. ** In one patient with favourable outcome to treatment, species was not mentioned in the journal of the patient; therefore, N = 52 for favourable response to treatment in this case.

## Data Availability

Due to privacy issues and in accordance with consent provided by participants regarding the use of confidential data, case-based data cannot be shared. Aggregated data can be shared upon request.
